# Impact of Volunteerism on the Mental Health and Academic Performance of Medical Students: A Saudi-Based Cross-Sectional Study

**DOI:** 10.7759/cureus.68855

**Published:** 2024-09-07

**Authors:** Saleh A Alghamdi, Abdulaziz T Alshomrani

**Affiliations:** 1 Department of Psychiatry, Imam Mohammad Ibn Saud Islamic University, Riyadh, SAU; 2 Department of Internal Medicine, College of Medicine, University of Bisha, Bisha, SAU

**Keywords:** anxiety, depression, medical students, mental health, volunteering activities

## Abstract

Introduction

The term "volunteering" refers to any endeavor in which one's time is devoted voluntarily to the benefit of another individual, group, or organization, and without the expectation of receiving compensation. Several studies have discovered that engagement in volunteer work is substantially predictive of improved mental and physical health, self-esteem, diminished depressive symptoms, and psychological distress, in addition to having a positive correlation with mental well-being.

Aim

The objective of this study was to investigate the correlation between medical student participation in volunteer work and their mental well-being.

Subject and methods

A cross-sectional survey was carried out in Riyadh, Saudi Arabia, involving medical students enrolled in Princess Nourah bint Abdulrahman University and Imam Mohammad Ibn Saud Islamic University, both of which are government institutions, as well as Almarefah College, a private medical school. The students were provided with a self-administered questionnaire. Sociodemographic information, prior volunteer experience, and an evaluation of the mental health status of medical students are all components of the questionnaire.

Results

A total of 827 medical students participated; 798 (96.5%) of them fell between 18 years and 25 years and only 29 (3.5%) were between 25 years and 40 years. The males accounted for 594 students (71.8%) and girls accounted for 233 students (28.2%). Among the students, 387 (46.8%) fell within the age range of 21 to 22 years, and 401 (48.5%) had a prior engagement in voluntary activities at school or college. Significant factors that influence participation in volunteering activities consist of attending a governmental institution, 616 students (74.5%); maintaining a grade point average (GPA) ranging from 2.75 to 3.74, 337 students (40.7%); and expressing a preference for participating in medical-related endeavors, 455 students (55%). There is no statistically significant correlation between medical students' engagement in volunteering activities and their mental health state (p>0.05).

Conclusion

A significant proportion of medical students actively engaged in volunteer work. Contrary to earlier findings, this study establishes that there is no correlation between engaging in volunteer work and the mental health conditions of medical students. Additional longitudinal studies are necessary to establish the correlation between engagement in volunteer work and the mental health of medical students.

## Introduction

Volunteering means any activity in which time is given freely to benefit another person, group, or organization [1] and providing a service without the intent of compensation [2].

Worldwide, the prevalence of adult volunteering varies considerably with estimates of 27% in the USA, 36% in Australia, and 22.5% in Europe (countries ranging from 10% as in Bulgaria, Greece, Italy, and Lithuania to >40% as in Austria, the Netherlands, Sweden and the UK) [3].

Volunteering is usually advantageous, although individuals' motivations for volunteering may differ based on several factors including socioeconomic situation, cultural background, personal experience, and other influences. A major motivator for young people is the opportunity to gain work-related experience, skills, and qualifications that can help them in their education and careers [4]. A research study from the United States found that volunteering can enhance students’ academic development, personal skills development, and sense of civic responsibility [5]. These career-related factors exist alongside a variety of other motivations and benefits. A national study of university students in England found that respondents gave both altruistic and instrumental reasons for volunteering [6]. Smith et al. showed that the most important reasons for volunteering were as follows: to help someone in their community; to learn new skills; to respond to their needs or skills; and, to help gain experience to benefit their future career [6].

Moreover, volunteering has been advocated by the United Nations, and American and European governments as a way to engage people in their local communities and improve social capital, with the potential for public health benefits such as improving wellbeing and decreasing health inequalities [3]. It has been found that volunteering has a positive relationship with health outcomes such as mental well-being [7], and is significantly predictive of better mental and physical health [8,9], self-esteem [10,11], lower depressive symptoms [9,12] and psychological distress [8,13]. The causal mechanisms remain unclear, but it has been hypothesized that volunteering would enhance well-being because it boosts self-esteem and builds social resources that in turn counteract the negative moods of anxiety and depression. While the studies provide evidence that volunteering can have favorable effects on mental health, they do not specify the specific motives or types of volunteering activities that yield these benefits. The majority of studies conducted on the relationship between volunteering and mental health have primarily focused on older age groups and overall mental well-being. The correlation between mental health in the young age group and volunteering remains little researched., although one study has found no association between volunteering and depression in the younger age group [13,14]. Moreover, there is a significant lack of research on the effects of volunteering on anxiety in comparison to the limited studies on the influence of volunteering on depression, which are also scarce.

While engaging in volunteer work is crucial for the development of medical students on a personal and professional level, it also entails several obstacles that can hinder students' commitment and have an impact on the overall experience, for example, the lack of awareness, not knowing how to volunteer, not being able to afford time off work, not being allowed time off work, inconvenient timing of sessions, not being able to commit long term, and discomfort with student touch [6]. Mawarni et al. examined numerous challenges that students may encounter during their participation in volunteer work, for example, uncontrollable turnover of volunteers and inconsistency in their commitment to the projects, facilitating students to determine the professional identity they want to focus on and managing work in a team with different backgrounds member [15].

In Saudi Arabia, the practice of volunteering has significantly grown in the past 30 years. The Saudi government enacted the Law of Associations and Civil Society Institutions on January 22, 1990, which encompasses volunteer work [16]. Subsequently, on January 22, 2020, the Voluntary Service Law was released [16]. In recent years, Saudi Arabian medical colleges have begun to actively promote student participation in volunteer services as an integral part of their curricula. To the best of our knowledge, there have been no studies investigating the correlation between Saudi medical students volunteering and their mental well-being, specifically in connection to depression and anxiety. This study aims to investigate the influence of volunteering on medical students’ anxiety and depression in Saudi Arabia and its impact on their academic performance.

## Materials and methods

Study design

This is a descriptive cross-sectional study that was conducted in Saudi Arabia, Riyadh City in January 2016. A paper-based questionnaire was distributed manually to a convenient cross-sectional population of students from two governmental medical schools, Imam Mohammad Ibn Saud Islamic University and Princess Nourah bint Abdulrahman University, as well as one private medical school, Almarefah College, all located in Riyadh.

Ethical consideration

Based on the Declaration of Helsinki, Institutional Review Board approval was obtained from the Medical Research Unit, College of Medicine, Imam Mohammad Ibn Saud Islamic University, Riyadh, Saudi Arabia, with Institutional Review Board number 0038/5/2016-56. Participation in this study was completely voluntary; each participant was notified of their consent and invited to participate. Participants did not earn a material income due to their participation.

Participation in the questionnaire was voluntary, and no incentives or guarantees were offered. This was clearly addressed during the data collection process. The academic personnel had no role in distributing or collecting the questionnaire. The initial statement of the survey assured the confidentiality of the participants, which was confirmed by refraining from asking any questions that could potentially disclose their identity, either directly or indirectly.

Study criteria

Inclusion criteria included any medical student, regardless of gender, who was enrolled in the three universities mentioned above. Any student who was not a medical student, failed to complete the questionnaire, or signed the consent form was excluded from the study.

Procedure

We developed a questionnaire that commences with an introductory statement that clarifies the purpose of the questionnaire, the expected time needed to complete it, and the goal of the study. The instructions clearly stated that completing the questionnaire implies giving agreement for the use of the collected data for research purposes, both in written and verbal form. 

After conducting a literature review and numerous discussion meetings, we developed a questionnaire comprising socio-demographic information, prior volunteer experience, and included Patient Health Questionnaire-4 (PHQ-4) to evaluate the mental health status of medical students [17].

Since the questionnaire (except for PHQ-4) was created by the researchers themselves and for face validity to measure what was intended to be measured, the preliminary questionnaire was submitted for evaluation to two experts in research methodology, and their recommendations were addressed. Following that, we administered the survey to a pilot group consisting of ten students, wherein we considered their feedback regarding the tool's clarity, difficulties, editing, and layout.

The researchers reassessed the participants' responses and feedback and made revisions and slight rephrasing to the questionnaire to make sure that it was clear to the respondents. 

Data collection tool

Each of the universities had a student representative who supervised and coordinated the distribution and collection of questionnaires among their colleagues. The distribution and collection of questionnaires took place over a period of four weeks, with each academic level being targeted at a separate time to accommodate their academic commitments. A portion of the students completed the questionnaire immediately, while others preferred to take it home and return it on another day.

The PHQ-4 has been used to measure the mental health condition of medical students. This is a four-item questionnaire with 4-point Likert scale categories ranging from "not at all" coded with 0 to "nearly every day" coded with 3. The first two questions measure the patients' anxiety, and the other two measure the depression levels. A score of 3 or higher indicates a mental health condition. However, PHQ is a valid and reliable tool to screen for depression and anxiety in a Saudi sample [17].

Statistical analysis

The data were analyzed using the software program IBM SPSS Statistics for Windows, Version 26 (IBM Corp., Armonk, NY, USA). Descriptive statistics were given as numbers and percentages (%) for all categorical variables. The relationship between participation in volunteering activities according to the medical students' socio-demographic characteristics, previous experience in volunteering activities, and mental health condition has been conducted using the Chi-square test. Based on the significant results, a multivariate regression analysis was subsequently performed to determine the significant independent predictors associated with volunteering activities with corresponding odds ratios and 95% confidence interval. Values were considered significant with a p-value of less than 0.05.

## Results

In January 2016, a survey was conducted through a population of 1,200 students, comprising both male and female individuals from both senior and junior levels. The study had a response rate of 63.6%, with a total of 827 medical students participating. Among the participants, 594 (71.8%) were males and 233 (28.2%) were females. A total of 387 (46.8%) individuals were between the ages of 21 and 22. A total of 401 individuals (48.5% of participants) had previously participated in voluntary activities at school or college, and 616 (74.5%) of the students belonged to a government college. The socio-demographic characteristics of the medical students are illustrated in detail in Table 1.

**Table 1:** Socio-demographic characteristics of the medical students (n=827) GPA: Grade Point Average

Study data	N (%)
Age group	
18 – 20 years	172 (20.8%)
21 – 22 years	387 (46.8%)
23 – 25 years	239 (28.9%)
>25 years	29 (03.5%)
Gender	
Male	594 (71.8%)
Female	233 (28.2%)
Marital status	
Single	775 (93.7%)
Married	49 (05.9%)
Divorced	02 (0.20%)
Widowed	01 (0.10%)
Residency status	
With the family	683 (82.6%)
With a relative	16 (01.9%)
With friends	31 (03.7%)
Alone	57 (06.9%)
University dormitory	40 (04.8%)
Type of university	
Governmental	616 (74.5%)
Private	211 (25.5%)
Academic year level	
Preparatory year	16 (01.9%)
1st year	180 (21.8%)
2nd year	179 (21.6%)
3rd Year	189 (22.9%)
4th year	164 (19.8%)
5th year	99 (12.0%)
GPA (out of 5)	
<2 (F)	06 (0.70%)
2 – 2.74 (D)	78 (09.4%)
2.75 – 3.74 (C)	337 (40.7%)
3.75 – 4.49 (B)	271 (32.8%)
4.5 – 5 (A)	135 (16.3%)

A total of 455 (55%) of the students preferred medical field volunteering activities and almost all students; 767 students (92.7%) denied negative reflections of their participation on their academic Grade Point Average (GPA). According to the PHQ-4 scale scores, 231 (29.9%) of the respondents may have a generalized anxiety disorder, while 274 (33.1%) of them may have depression. Table 2 summarizes details related to students' previous volunteering, preferences, reflection on their GPA, and families' attitudes. 

**Table 2:** Previous volunteering, preferences, reflection on GPA and mental health (n=827) † Variable with multiple response answers PHQ-4: Patient Health Questionnaire-4, GPA: Grade Point Average

Study data	N (%)
Have you ever participated in volunteering activities?	
Yes	401 (48.5%)
No	426 (51.5%)
If the answer is yes, how many participations? ^(n=401)^	
1-2	133 (33.2%)
3-4	149 (37.2%)
5-6	53 (13.2%)
>6	66 (16.5%)
When did you start volunteering? ^(n=401)^	
Elementary school	41 (10.2%)
Intermediate school	56 (14.0%)
High school	94 (23.4%)
University	159 (39.7%)
Not mentioned	51 (12.7%)
Are you a member of a volunteering organization?	
Yes	111 (13.4%)
No	716 (86.6%)
What type of volunteering activity do you like? ^†^	
Medical	455 (55.0%)
Social	211 (25.5%)
Psychological	82 (09.9%)
Religious	72 (08.7%)
Sports	60 (07.3%)
Others	02 (0.20%)
Was your academic performance affected by your volunteering activities? Effect on GPA.	
Yes	60 (07.3%)
No	767 (92.7%)
What are the preferred times for you to volunteer?	
On vacations	300 (36.3%)
During the academic year	251 (30.4%)
On religious or social occasions	83 (10.0%)
Not mentioned	193 (23.3%)
Generalized anxiety disorder (PHQ-4)	
Yes	231 (29.9%)
No	596 (70.1%)
Depression (PHQ-4)	
Yes	274 (33.1%)
No	535 (66.9%)

**Figure 1:**
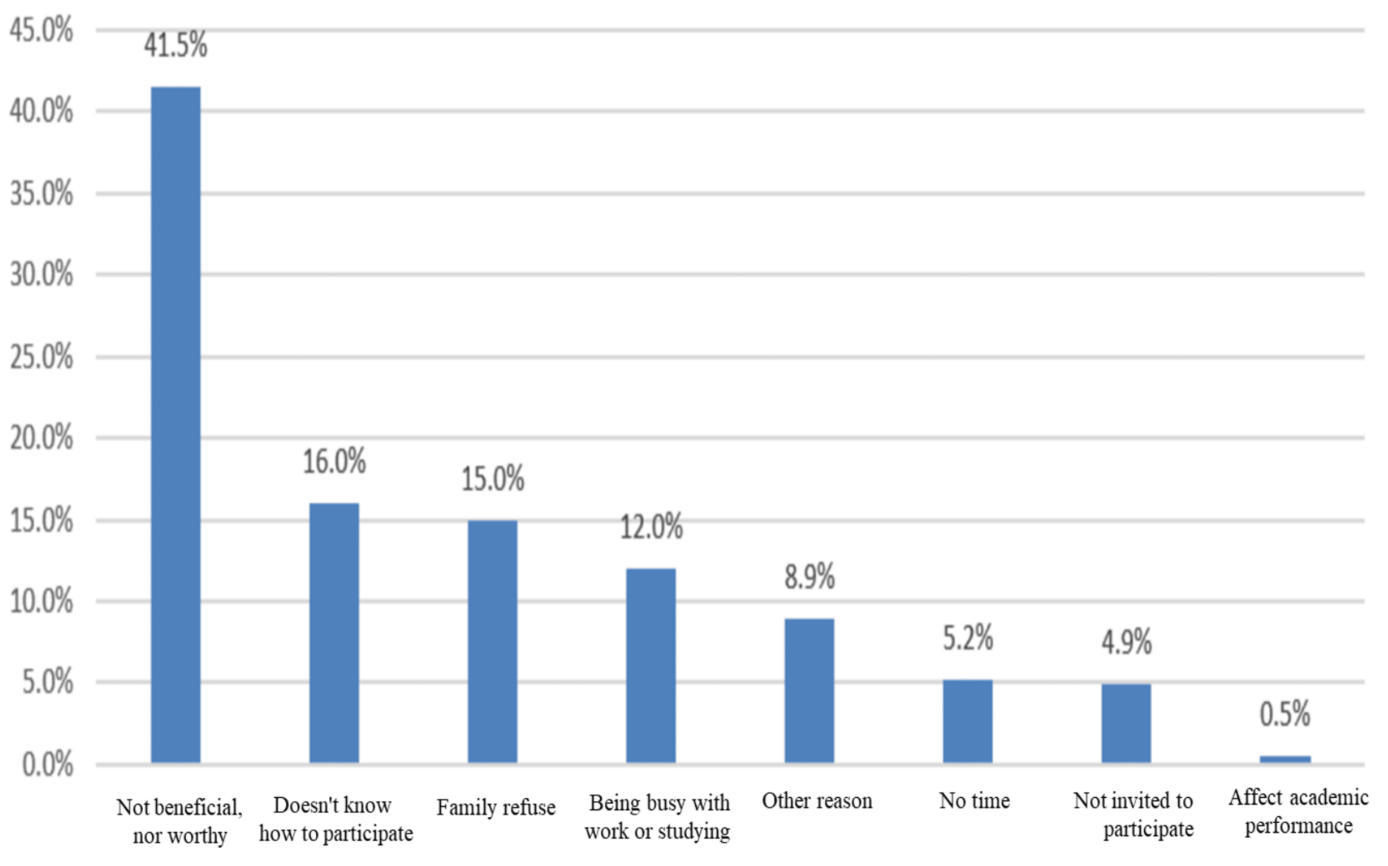
Barriers to participate in volunteering activities

The survey showed that among those who did not participate in volunteering activities; 426 students (51.5%), the most common barrier to volunteering was the belief that volunteering is not beneficial nor worthy as reported by 177 (41.5%) students, followed by the lack of information on how to participate by 68 (16%) student, family refusal by 64 (15%) student, and being busy by work or studying by 51 (12%) student as shown in Figure 1.

**Figure 2:**
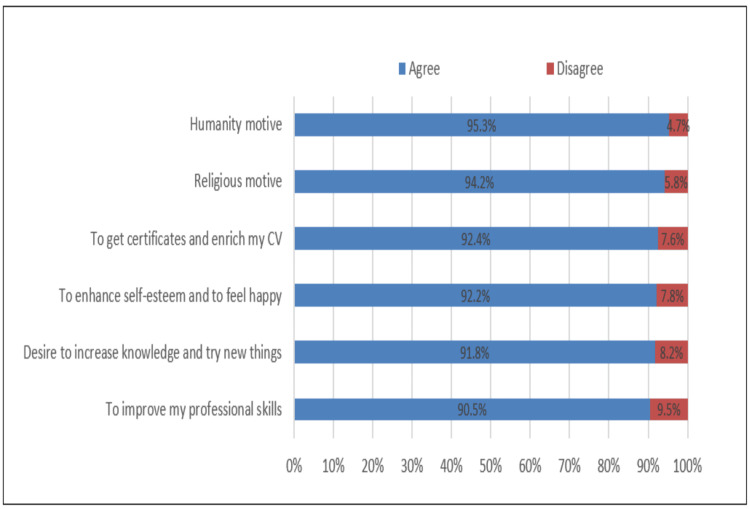
Motives toward participating in volunteering activities

As shown in Figure 2, the major motivators for volunteering were humanitarian as reported by 406 (95.3%) students; religious, by 401 (94.2%) students; getting certificates and enriching their curriculum vitae, by 394 (92.4) students; well-being, by 393 (92.2%) students; curiosity (91.8%); and skills improvement by 391 (90.5%) students.

**Figure 3:**
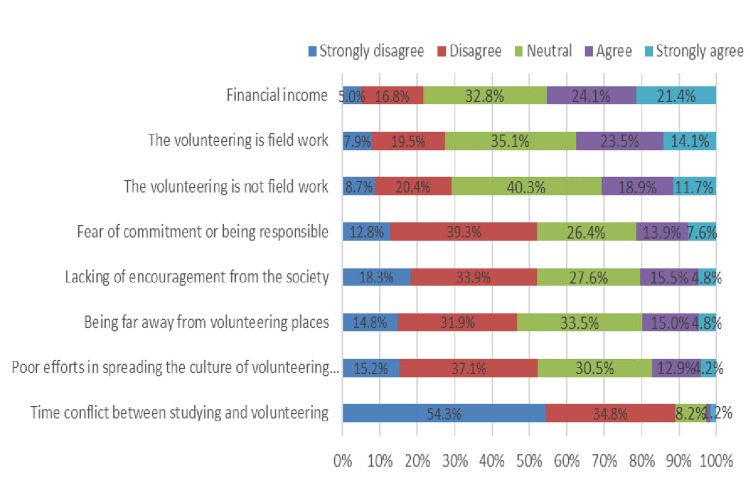
Encountered challenges during participation in volunteering activities

Participants strongly agreed that the most common challenges encountered during volunteering activities were financial as reported by 194 (45.5%) students, and the lowest challenges were the time conflict between studying and volunteering as reported by 43 (10.2%) students. Figure 3 summarizes participants' responses related to volunteering challenges.

However, it is important to note that our study showed that the most common barrier to volunteering was the belief that volunteering is not beneficial nor worthy (41.5%); this was followed by the lack of information on how to participate (16%), family refusal (15%), and being occupied by work or studying (12%).

**Table 3:** Relationship between participation in volunteering activities according to students' socio-demographic characteristics, previous experience in volunteering, and mental health conditions (n=827) † Variable with multiple response answers § P-value has been calculated using the Chi-square test ** Significant at p<0.05 level GPA: Grade Point Average

Factor	Participated in volunteering activities		P-value ^§^
	Yes N (%) ^(n=401)^	No N (%) ^(n=426)^	
Age group			
<22 years	172 (42.9%)	182 (42.7%)	0.961
≥22 years	229 (57.1%)	244 (57.3%)	
Gender			
Male	271 (67.6%)	323 (75.8%)	0.008 **
Female	130 (32.4%)	103 (24.2%)	
Residency status			
Living with family	345 (86.0%)	338 (79.3%)	0.011 **
Not living with family	56 (14.0%)	88 (20.7%)	
Type of university			
Governmental	347 (86.5%)	269 (63.1%)	<0.001 **
Private	54 (13.5%)	157 (36.9%)	
Academic year level			
Junior students (Prep – 2^nd^ year)	168 (41.9%)	207 (48.6%)	0.053
Senior student (3^rd^ – 5^th^ year)	233 (58.1%)	219 (51.4%)	
GPA (out of 5)			
<2.75	21 (52.1%)	63 (14.8%)	<0.001 **
2.75 – 3.74	137 (34.2%)	200 (46.9%)	
3.75 – 4.49	166 (41.4%)	105 (24.6%)	
4.5 – 5	77 (19.2%)	58 (13.6%)	
Member of volunteering organization			
Yes	92 (22.9%)	19 (04.5%)	<0.001 **
No	309 (77.1%)	407 (95.5%)	
Academic performance affected by volunteering activities			
Yes	40 (10.0%)	20 (04.7%)	0.003 **
No	361 (90.0%)	406 (95.3%)	
Where do you prefer to volunteer? ^†^			
Inside a college or university	187 (46.6%)	55 (12.9%)	<0.001 **
Malls	176 (43.9%)	69 (16.2%)	<0.001 **
Conferences	187 (46.6%)	73 (17.1%)	<0.001 **
Outside city	50 (12.5%)	18 (04.2%)	<0.001 **
Outside country	72 (18.0%)	32 (07.5%)	<0.001 **
Preferred type of volunteering activity ^†^			
Religious	48 (12.0%)	24 (05.6%)	0.001 **
Medical	315 (78.6%)	140 (32.9%)	<0.001 **
Social	143 (35.7%)	68 (16.0%)	<0.001 **
Psychological	57 (14.2%)	25 (05.9%)	<0.001 **
Sports	44 (11.0%)	16 (03.8%)	<0.001 **
Symptoms of anxiety			
Yes	120 (29.9%)	111 (26.1%)	0.215
No	281 (70.1%)	315 (73.9%)	
Symptoms of depression			
Yes	138 (34.4%)	136 (31.9%)	0.447
No	263 (65.6%)	290 (68.1%)	

When measuring the relationship between previous participation in volunteering activities among the socio-demographic characteristics, previous experience in volunteering activities, and mental health condition of the medical students (Table 3), it was observed that the prevalence of medical students who had previous volunteering experience was significantly more common among female gender (p=0.008), those living with their family (p=0.011), those who were studying at governmental school (p<0.001), those who had GPA rating between 3.75 and 4.49 (p<0.001), those who were a member of volunteering organization (p<0.001), those who their academic performance were affected due to volunteering activities (p=0.003), those who preferred to participate in volunteering activities inside college or university (p<0.001), malls (p<0.001), conferences (p<0.001), outside city (p<0.001) and outside country (p<0.001), and those who preferred voluntary activity through religious activities (p=0.001), medical (p<0.001), social (p<0.001), psychological (p<0.001) and sports (p<0.001). Furthermore, Table 3 shows that the correlation between participation in volunteering work and the mental health condition (depression and anxiety) of the medical students was insignificant.

**Table 4:** Multivariate regression analysis to determine whether previous participation in volunteering activities has a significant impact psychologically or with specific demographic profiles (n=827) † Variable with multiple response answers ** Significant at p<0.05 level. AOR: adjusted odds ratio; CI: confidence interval; GPA: Grade Point Average

Factor	AOR	95% CI	P-value
Gender			
Male	Ref		
Female	1.219	0.781 – 1.901	0.383
Residency status			
Living with family	Ref		
Not living with family	1.052	0.647 – 1.710	0.838
Type of university			
Governmental	1.945	1.202 – 3.148	0.007 **
Private	Ref		
GPA (out of 5)			
<2.75	Ref		
2.75 – 3.74	2.455	1.164 – 5.178	0.018 **
3.75 – 4.49	1.259	0.745 – 2.128	0.389
4.5 – 5	0.658	0.395 – 1.094	0.107
Member of volunteering organization			
Yes	3.153	1.761 – 5.646	<0.001 **
No	Ref		
Preferred type of volunteering activity ^†^			
Religious	0.791	0.427 – 1.468	0.458
Medical	2.344	1.495 – 3.675	<0.001 **
Social	1.036	0.651 – 1.649	0.881
Psychological	0.831	0.438 – 1.578	0.572
Sports	1.162	0.571 – 2.362	0.679

Based on significant results, a multivariate regression analysis was subsequently performed in Table 4 to determine the independent, influential predictors of previous participation in volunteering activities. Based on the results it was observed that studying at a governmental institution, having the lowest GPA ratings, being a member of a volunteering organization, preferring to participate in volunteering activities through conferences, and participating in religious-type volunteering activities were the significant independent predictors of increased participation to volunteering activities, while preference to volunteering activities inside a college/university or inside malls were the significant independent predictors of decreased participation in volunteering activities. This further means that compared to students enrolled in a private institution, students who were enrolled in governmental institutions were at increased odds of participating in volunteering activities by at least 1.94 times higher (AOR=1.945; 95% CI=1.202 - 3.148; p=0.007). Compared to students with the lowest GPA, students who had a GPA between 2.75 and 3.74 were at increased odds of participating in volunteering activities by at least 2.46-fold higher (AOR=2.455; 95% CI=1.164 - 5.178; p=0.018). Students who were members of volunteering organizations were 3.15 times more likely to participate in volunteering activities (AOR=3.153; 95% CI=1.761 - 5.646; p<0.001). Students who preferred to participate in voluntary activities in conferences were 1.60 times more likely to participate in volunteering activities (AOR=1.605; 95% CI=1.033 - 2.494; p=0.035). Students who preferred to volunteer in medical activities were 2.34 times more likely to participate in volunteering activities (AOR=2.344; 95% CI=1.495 - 3.675). On the contrary, students who preferred to participate in voluntary activities inside a college or university were at decreased odds of participating in actual voluntary activities by at least 63% (AOR=0.370; 95% CI=0.238 - 0.575; p<0.001), while those who preferred to volunteer in malls were also at decreased odds by at least 60% (AOR=0.591; 95% CI=0.388 - 0.900; p=0.014).

Data from our study showed that volunteering activities were influenced by female students, those who lived with their families, those who were studying in government institutions, those who had GPA (2.75-3.74) ratings, those who were members of volunteering organizations, and those who indicated that their academic performance was affected by the volunteering activities. However, in our adjusted regression estimates, only students in government institutions, GPA (2.75-3.74) ratings, and being a member of a volunteering organization remained significant and determined as the significant independent predictors of willingness to participate in volunteering activities.

## Discussion

Data from our study showed that almost half of medical students have participated in volunteering activities, and more than two-thirds of them reported three to four volunteering time at least. These percentages are higher than American and European adult general population statistics [3], however, it is difficult to compare them with our numbers due to studies' population differences and the nature of volunteering behavior definitions. Humanitarian, religious, professional career development, well-being, curiosity, and skills improvement scored high as a motivator for volunteering and no single motivator showed a significant difference which reflects the multipurpose nature of volunteering. These motivators are not much different from what is mentioned in other studies. Humanitarian -as in our studies-, moral, or to help those in need were the most consistent frequent motivators for volunteering in most studies [6,18,19]. In addition, professional career development is addressed very often as a major motivator. Reflection on well-being or satisfactory feelings is not an uncommon motivation factor [20]. Religion was a major motivator in Saudi medical students, however, this purpose is addressed in other studies to a lesser degree in comparison to the aforementioned motivators [20] and may be excluded as a motivator in some countries like Poland [21], which may be attributed to cultural differences and attitude toward religion.

The most common barrier to volunteering in our study is the lack of interest. However, few studies addressed the same reason not to such an extent [22,23]. This issue should be addressed in the college curriculum, student assessments, more education, orientations, motivation enhancement, and academic, and extracurricular activities. Another interesting barrier was the lack of knowledge of how to participate which was admitted as a barrier in some other studies [22,23]. This emphasizes the role of the university, college administration, students' affairs, mentors, and the students' club to make students' involvement simple, obvious, and easy. Although students' concerns about the conflicts between volunteering and academic achievement were expected as in some other studies, however only less than 1% of respondents affirm that concern which may pave the way for more students to indulge in volunteering.

Fair academic performance (GPA 2.75-3.74) is associated more with previous volunteering in comparison to high or low GPA, although most studies admit an association between volunteering and high GPA [20,24,25]. No marked justification for the inconsistent finding which may need further exploration in new studies. The gender showed no significant differences in volunteering in comparison to most of the studies that found female students have more volunteering participation -other than COVID-19-related studies [18,24,26].

Regarding mental health, our findings showed no marked association between volunteering, depression, and anxiety scorings. Most studies that study volunteering and mental health admit the association [8,15]. Although it needs further study and exploration, scales and mental health addressed issues may explain in part these differences.

Study limitations

Although our study examines a substantial domain that has been inadequately explored in Saudi Arabia, it is not devoid of limitations. Self-reported data used in cross-sectional studies employing convenient sampling methods are susceptible to bias and misinterpretations, both of which may significantly influence the results of the research. In addition, since the questionnaire (except of PHQ-4) was created by the researchers themselves, it lacked the characteristics of a standardized, well-established instrument. Despite the fact that the PHQ-4 is a validated screening tool for depression and anxiety, a clinical assessment is required to establish a clinical diagnosis. On account of the fact that the results were derived from a survey of medical students from Riyadh, Saudi Arabia only, the study's applicability was restricted.

## Conclusions

Medical students are highly eager to engage in voluntary service. Students attending government universities with lower Grade Point Averages and affiliated with volunteer organizations exhibited a higher propensity for engaging in volunteer activities compared to other medical students. Nevertheless, the engagement in volunteer work did not appear to have an impact on the mental well-being of our population. The primary motivations were regarded to be humanity and religion, whereas financial income and fieldwork volunteering posed a particular obstacle. Notably, almost half of the medical students believed that engaging in volunteer work did not provide any advantages. It is imperative to incorporate volunteering programs into the university curriculum. Participation in voluntary work throughout medical school allows for the exploration of the personality qualities of the students.
